# Evaluating the reliability and validity of a Chinese version of the performance-oriented mobility assessment among patients with chronic stroke

**DOI:** 10.3389/fneur.2024.1461069

**Published:** 2024-11-13

**Authors:** Ming Zhong, Yongnan Jing, Xiaofeng Zhao, Ying Gao, Yanju Jiang, Yihao Liu, Chunping Du

**Affiliations:** ^1^Department of Sports Medicine, Sichuan Provincial Orthopedics Hospital, Chengdu, China; ^2^Department of Rehabilitation, Shanghai MCC Hospital, Shanghai, China; ^3^School of Acupuncture-Moxibustion and Tuina and School of Health Preservation and Rehabilitation, Nanjing University of Chinese Medicine, Nanjing, China; ^4^School of Psychology, Faculty of Health and Life Sciences, University of Exeter, Exeter, United Kingdom; ^5^Rehabilitation Medicine Center and Institute of Rehabilitation Medicine, West China Hospital, Sichuan University, Chengdu, China; ^6^Key Laboratory of Rehabilitation Medicine in Sichuan Province, West China Hospital, Sichuan University, Chengdu, China

**Keywords:** stroke, performance-oriented mobility assessment, risk-of-fall, balance, gait, reliability

## Abstract

**Background:**

The Performance-Oriented Mobility Assessment (POMA) is a reliable instrument for evaluating the mobility (balance and gait) of patients with chronic stroke to manage their risk of falling; however, it has not been validated among Chinese patients with stroke. This study aimed to evaluate the reliability and validity of the Chinese POMA in patients with stroke.

**Methods:**

The POMA was applied to volunteer patients with stroke from the Shanghai MCC Hospital. The patients underwent the Chinese POMA, Berg balance scale (BBS), and timed up and go (TUG) tests on the first day of inpatient treatment. The same physician repeated the tests the next day to assess test–retest reliability, and upon the patient’s discharge from the inpatient department, two different physicians measured inter-rater reliability.

**Results:**

The study involved 76 patients with stroke (age: 62.04 ± 9.76 years; 34.2% female). The results showed that the Chinese POMA had good overall internal consistency (*σ*=. 875), with a moderate consistency between its two subscales (balance *σ* = 0.875; gait *σ* = 0.668). The individual items showed high test–retest (ICC = 0.997) and inter-rater reliability (ICC = 0.988). The content validity test showed high correlations between the Chinese POMA, the BBS (rs = 0.70), and the TUG (rs = −0.75). However, the confirmatory factor analysis suggested that the two-factor model (balance and gait) was mediocre.

**Conclusion:**

The Chinese POMA showed acceptable reliability and validity for evaluating mobility (balance and gait) in Chinese patients with stroke in terms of their risk of falling. However, further evaluation of the two-factor model (balance and gait) is required.

## 1 Introduction

According to the latest reports estimating the global prevalence of stroke, the age-standardized prevalence of stroke in China is estimated to be 2% among the Chinese population, accounting for 40% of the world’s stroke cases (1), and the incidence rate is estimated to be 14.48%. This indicates approximately 28 million active stroke cases in China that need treatment and medical care. One of the risk factors for stroke mortality is the risk of falls because patients with stroke often have impaired motor function (2). Consequently, instruments are required to assess mobility and manage the risk of falls in Chinese patients with stroke.

The Performance-Oriented Mobility Assessment (POMA) is suggested to be a reliable instrument for mobility (balance and gait) among patients with stroke (3); it was initially designed to measure mobility (balance and gait) among the elderly (4, 5). The POMA has also been translated and validated in other languages (6, 7). A validation investigation of Turkish and Persian POMA translations was conducted among healthy elderly individuals and yielded high reliability and validity. In both reliability and validity studies, the POMA was measured for internal consistency, test–retest reliability, inter-rater reliability, and content validity in correlation with the Berg Balance Scale (BBS) (8) and the Timed Up and Go test (TUG) (9). Specifically, the BBS is considered a useful instrument for measuring balance ability in patients with stroke (3, 10), and the TUG test is considered to reflect the functional ability and risk of falls in patients with stroke (3, 11). Consequently, these two measurements have been used in previous reliability studies of the POMA as an external reference for content validity.

The POMA has been widely used and translated into many languages and has been recommended by the Chinese Medicine Association to assess mobility for Chinese seniors (12). A formal validation of the Chinese POMA has not been conducted for patients with chronic stroke, which could be a potentially helpful instrument for determining the prevalence of Chinese patients with stroke. The current study aimed to translate the POMA into Chinese and evaluate its reliability and validity using a method similar to that used in relevant research.

## 2 Method

### 2.1 Participants

The inclusion criteria were participants who were (1) native Mandarin Chinese speakers, (2) diagnosed with chronic stroke based on brain imaging, (3) able to comprehend the (simplified) Chinese semantic context, and (4) able to walk with aid. The exclusion criteria were participants who (1) had any self-reported history of comorbidities that could not be mobilized, such as osteoarthritis, cerebellar atrophy, or heart disease, or (2) had hearing or cognitive impairment, including those with language comprehension screened with the post-stroke language assessment sets (13). The participants signed up for the experiment as volunteers and did not receive any payment for their participation.

### 2.2 Procedure and material

The procedure of the current experiment was similar to that of a previous study evaluating the reliability of the POMA among patients with stroke (3), following the COSMIN checklist. The target sample size was estimated using the formula by Bonett (14):

n=8×z∝22×1−ρ˜I2×1+k−1×ρ˜I2k×k−1×w2+1

Where (1) *n* is the target sample size; (2) *σ* is the significance level (default = 0.05); (3) z_σ/2_^2^ is the point on a standard normal distribution exceeded with probability at σ/2 (fixed at 1.96^2^); (4) $\tilde{\rho }$ is the expected intraclass correlation coefficient (ICC); (5) *k* is the number of instrument scales; (6) *w* is the desired tolerance width. The calculation was conducted using an online sample size calculator developed for reliability studies (https://wnarifin.github.io/ssc/ssicc.html) based on the above formula (15). Accordingly, previous studies suggested the ICC ranged from 0.75 to 0.97 (6, 7), with no estimated dropping rate. Consequently, we would expect a similar ICC of approximately 0.85 with a 0.1 tolerance width. The calculator estimated a minimum sample size of 23 using the 3 instruments used in the current study. However, validation studies of questionnaires generally require a sample of 5 or 10 times the number of items. In this case, evaluating the 16-item POMA is expected to reach at least a sample of 80, which would be our target.

The participants were recruited from the Rehabilitation Department at Shanghai MCC Hospital based on the inclusion criteria, and they consented and were screened for eligibility by three physiatrists based on the exclusion criteria. Inpatients were registered with a code for their individual hospital beds at administration, and this code was used in the current study to identify participants’ data. The physicians contacted the participants through their bedcode because there were multiple waves of measurements in the current study. Consequently, the researchers were blinded when accessing the data through bed codes and could not access the participants’ personal information during or after the experiment.

The physicians conducting the ratings underwent a structured training program to ensure accurate and consistent administration of the Chinese POMA. The training began with a comprehensive introduction to the assessment tool, including detailed explanations of the balance and gait items. The physicians were then trained to administer each item task step-by-step, led by a senior physiotherapist (YJ). The physicians participated in practical demonstrations by the senior physiotherapist administering the assessment on patients, followed by hands-on practice. During this practice, each physician administered the POMA to individuals simulating stroke-related mobility impairments, receiving real-time feedback from the senior physiotherapist. The focus of the training was on understanding each item’s criteria, correct task execution, and unbiased, objective scoring. Once the physicians and experiment settings were well-prepared, eligible participants underwent the measurements in the following order:

The participants’ gait and balance were measured using the Chinese POMA on the first day of admission to the inpatient department. The Chinese POMA preserves the same structure as the original English version, containing 16 items assessing participants’ gait and balance. The POMA ranges from 0 to 28, including seven items assessing gait ranging from 0 to 12 and nine items assessing balance ranging from 0 to 16. A score of <24 indicates a potential impairment in balance, and a score of <15 indicates a potential risk of falls. An independent physiatrist conducted the assessment by instructing the participants to perform movements on the POMA and scored them from 0 to 2 based on the participants’ performance. The participants rested for 10–15 min after each assessment. The Chinese POMA and the original English versions are attached in the Supplementary material.

Participants were then assessed using the BBS to re-evaluate balance by another independent physiatrist. The BBS consists of 14 items scored from 1 to 4 to assess balance in the elderly with good reliability (8, 16). The scale scores ranged from 0 to 56. Scoring from 0 to 20 indicates poor balance, potentially wheelchaired, and the risk of falls; scoring from 21 to 40 indicates some balance to mobilize with aid and the risk of falls; and scoring from 41 to 56 indicates a fair balance to mobilize without aid. An independent physiatrist instructed the participants to perform movements on the BBS and assessed their performance.

After the BBS, a third independent physiatrist measured the participants’ balance using the TUG test (9, 17). The TUG required the participants to stand up from an armed chair with back support; the chair was 46 cm high, with an arm 21 cm high. Participants were instructed to sit on a chair with arms resting on the chair arms and back supported, stand up upon instruction, walk toward a marked position 3 meters in front of the chair, and walk back to sit on the chair again. An independent physiatrist recorded the participants’ time spent on the test. The test was repeated three times with a 1 to 2-min break. The results are presented as the mean time spent on the three tests.

**Figure 1:**
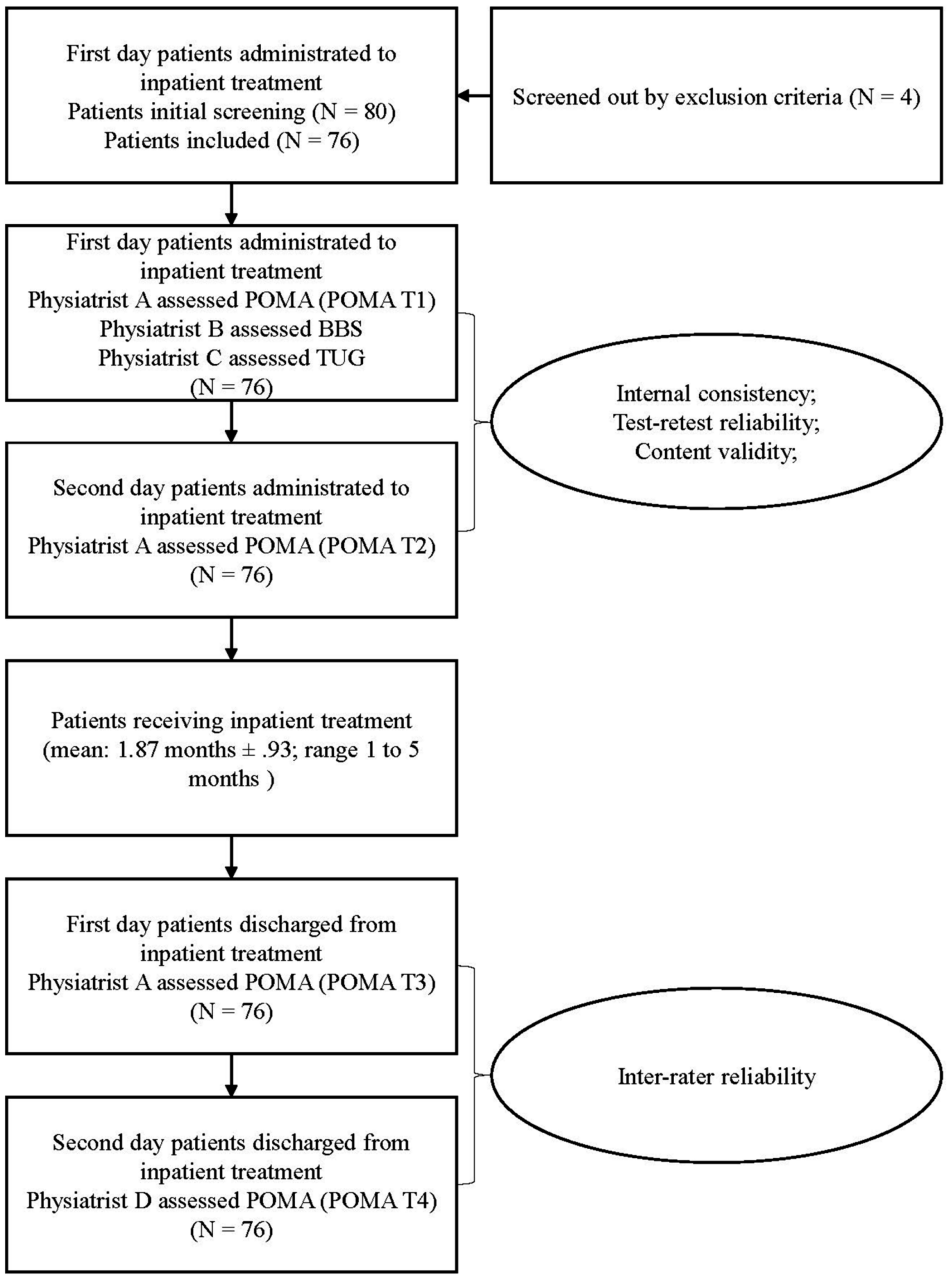
Experiment procedure flow.

Each set of ratings takes a long time and requires considerable effort from the patients. Considering ethics and clinical practice, it is impractical to measure the patients twice on the same day, either to rapidly conduct multiple trials in the upcoming days, because it may develop the practice effect that patients would improve their performance with rapid physical assessments (18, 19) or bias the consistency in reliability tests. The same physiatrist measured the Chinese POMA the next day to estimate the test–retest reliability. Test–retest reliability aims to determine the extent to which unchanged scores are the same for repeated measurements (20). However, since the patients were receiving inpatient treatment, it would have been impossible to compare the POMA results from baseline with the results after some period of time because the POMA outcomes would have been improved with the treatment they received. Therefore, the current test–retest reliability used the outcome from the second day of administration to inpatient treatment, which is consistent with another POMA reliability study (3). To determine the inter-rater reliability, two independent physiatrists measured the Chinese POMA again on the first and second days after the participants were discharged from inpatient treatment. Consequently, as summarized in Figure 1, the Chinese POMA was measured four times by two raters (POMA T1, T2, T3, and T4), where two were measured before (POMA T1 and T2) and two after inpatient treatment (POMA T3 and T4), and The BBS and TUG were measured on the first day of administration to the inpatient treatment (after POMA T1).

### 2.3 Statistical analysis plan

Statistical analyses were conducted in SPSS v29 with AMOS (IBM Corp., Armonk, NY, USA). The pilot data were entered into the reliability test estimated using Cronbach’s *α* coefficient to determine its internal consistency and Pearson bivariate correlation with BBS to check for its content validity quickly. Regarding the formal reliability test data, the individual POMA item ratings of four measurements were entered into the reliability test estimated using Cronbach’s α coefficient to determine its internal consistency. Then, the POMA ratings measured by the same physiatrists on the first and second days of patient admission to the inpatient department were entered into the test–retest reliability test estimated using the ICC (POMA T1 vs. T2). ICC absolute agreement was used because the current measurements obtained their own mean score (21) for patients with different stroke severities. The ICC of the test–retest reliability and the standard error of the mean (SEM) were used to calculate the minimal detectable change (MDC) at a 95% confidence interval using the following formula (22):

$${MDC}_{95\%}=1.96\times \sqrt{2}\times SEM$$

SEM was expressed using the following formula, where SDtrial 1 refers to the standard deviation of the first trial of POMA (POMA T1):

$$SEM={SD}_{\mathrm{trial}\;1}\times \sqrt{1-ICC}$$

The POMA ratings measured by two independent physiatrists on the first and second days after patient discharge from the inpatient department were entered into an inter-rater reliability test estimated using the ICC (POMA T3 vs. T4).

After evaluating the reliability, confirmatory factor analysis (CFA) was conducted to evaluate the validity of the two components (gait and balance) of the POMA using SPSS 29 AMOS. A lower Chi^2^ value indicates a better fit. A value of Root Mean Square Error of Approximation with the 90% confidence interval (RMSEA[90%]) ≤ 0.05 indicates a close fit, RMSEA [90%] between 0.05 and 0.08 indicates a reasonable fit, and RMSEA [90%] between 0.08 and 0.10 suggests a mediocre fit. RMSEA[90%] > 0.10, indicative of a poor fit. Comparative Fit Index (CFI) and Tucker-Lewis Index (TLI) close to 1 indicate a good fit, with values ≥0.95 suggesting an excellent fit and values between 0.90 and 0.95 indicating an acceptable fit. Finally, Standardized Root Mean Square Residual (SRMR) ≤ 0.08 indicates a good fit. A lower SRMR suggests a model that better reproduces the observed covariances.

Finally, the POMA results were entered into a Pearson bivariate correlation with the BBS and TUG results to evaluate external construct validity. A Cronbach’s *α* and ICC range from 0.6 to 0.75 is considered moderate, a Cronbach’s α and ICC range from 0.75 to 0.9 is considered good, and a Cronbach’s α and ICC greater than 0.9 is considered excellent (23, 24). Regarding the correlation between the Chinese POMA, BBS, and TUG, a coefficient range from 0.6 to 0.8 is considered strong, and a coefficient greater than 0.8 is considered very strong (25).

### 2.4 Ethics statement

The ethics application was submitted and approved by the ethics committee of the Shanghai MCC Hospital (Reference No: ZYLS202001), where the data was collected. Participants provided written informed consent before enrolment in the experiment.

## 3 Results

### 3.1 Participants characteristic information

Eighty participants who met the inclusion criteria were recruited. Four participants violated the exclusion criteria and were excluded from the study, leaving 76 eligible participants for analysis, including 50 men and 26 women (34.2% women). Among these, 28 had left hemiplegia and 48 had right hemiplegia. Detailed characteristics of the participants are presented in Table 1.

**Table 1:** Participants’ characteristic information.

	Mean (SD)	Range
Age	62.04 ± 9.76	29–82
Stroke duration	4.91 ± 2.65	1–12 (months)
Inpatient duration	1.87 ± 0.93	1–5 (months)
POMA T1	16.99 ± 5.45	4–27
POMA T2	17.05 ± 5.49	4–27
POMA T3	21.21 ± 5.72	4–28
POMA T4	21.12 ± 5.71	4–28

POMA T1 and POMA T2 refer to the POMA measured on the first and second day of administration to the inpatient, respectively, rated by the same physiatrist; POMA T3 and POMA T4 refer to the POMA measured on the first and second day of discharge from the inpatient by two independent physiatrists. Please contact the corresponding author for the detailed raw data of individual items.

### 3.2 Internal consistency

As shown in Table 2, the overall ratings of the Chinese POMA yielded Cronbach’s *α* from 0.875 to 0.901, suggesting a good internal consistency across four measurements. The balance subscale yielded Cronbach’s α ranged from 0.842 to 0.930, and gait subscales yielded Cronbach’s α ranged from 0.668 to 0.736, suggesting a moderate to good internal consistency across four ratings.

**Table 2:** Internal consistency of the Chinese POMA scale.

Chronbach’s σ	Day 1 on inpatient	Day 2 on inpatient	Day 1 post-inpatient	Day 2 post-inpatient
Balance	0.930	0.842	0.871	0.871
Gait	0.688	0.668	0.717	0.736
Overall	0.879	0.875	0.901	0.901

### 3.3 Test–retest reliability

As shown in Table 3, the overall POMA scale yielded excellent test–retest reliability (ICC = 0.997), with an MDC_95%_ of 1. The balance and gait subscale scores also yielded excellent test–retest reliability (ICC = 0.997, ICC = 0.993, respectively). The individual POMA items yielded a high ICC from 0.878 to 983, suggesting good-to-excellent test–retest reliability.

**Table 3:** Test–retest reliability test.

Item	POMA T1 mean	T1 SD	POMA T2 mean	T2 SD	Intraclass correlation coefficient	[95% CI]		MDC_95%_
Balance1	1.00	0.00	1.00	0.00	1.000			
Balance2	1.47	0.58	1.50	0.58	0.980	[0.969]	[0.987]	
Balance3	1.64	0.58	1.64	0.58	1.000			
Balance4	1.30	0.61	1.30	0.61	0.982	[0.971]	[0.988]	
Balance5	1.26	0.53	1.26	0.53	1.000			
Balance6	1.20	0.61	1.20	0.59	0.972	[0.975]	[0.983]	
Balance7	0.43	0.50	0.43	0.50	0.981	[0.970]	[0.988]	
Balance8	0.57	0.62	0.58	0.62	0.991	[0.986]	[0.994]	
Balance9	1.47	0.50	1.46	0.53	0.961	[0.939]	[0.975]	
Balance total	10.36	3.22	10.38	3.20	0.997	[0.995]	[0.998]	0.5
Gait1	0.83	0.38	0.83	0.38	1.000			
Gait2	2.84	1.11	2.82	1.13	0.978	[0.966]	[0.986]	
Gait3	0.14	0.35	0.17	0.47	0.872	[0.798]	[0.919]	
Gait4	0.38	0.49	0.41	0.50	0.942	[0.909]	[0.964]	
Gait5	1.16	0.59	1.16	0.59	0.980	[0.969]	[0.988]	
Gait6	1.12	0.65	1.12	0.65	0.984	[0.975]	[0.990]	
Gait7	0.16	0.37	0.17	0.41	0.878	[0.807]	[0.922]	
Gait total	6.63	2.54	6.67	2.57	0.993	[0.990]	[0.996]	0.5
POMA total	16.99	5.45	17.05	5.49	0.997	[0.996]	[0.998]	1

Test–retest reliability was conducted between the POMA ratings on the first and second days of patients’ administration to inpatient treatment by the same physiatrist (*N* = 76).

### 3.4 Inter-rater consistency

As shown in Table 4, the inter-rater reliability tests between the ratings from the two individual physiatrists 2 days after inpatient treatment yielded high intraclass correlation coefficients of the overall POMA ratings (ICC = 0.996), POMA balance subscale (ICC = 0.999) and POMA gait subscale (ICC = 0.988), suggesting a high (post-treatment) inter-rater consistency.

**Table 4:** Inter-rater reliability test.

Item	Inter-rater intraclass correlation coefficient [95% CI]		
Balance	0.996	[0.994]	[0.998]
Gait	0.999	[0.998]	[0.999]
Overall	0.988	[0.981]	[0.993]

The inter-rater reliability test was conducted on the first and second days of patients’ discharge from inpatient treatment by two independent physiatrists (*N* = 76).

### 3.5 Confirmatory factor analysis

**Figure 2:**
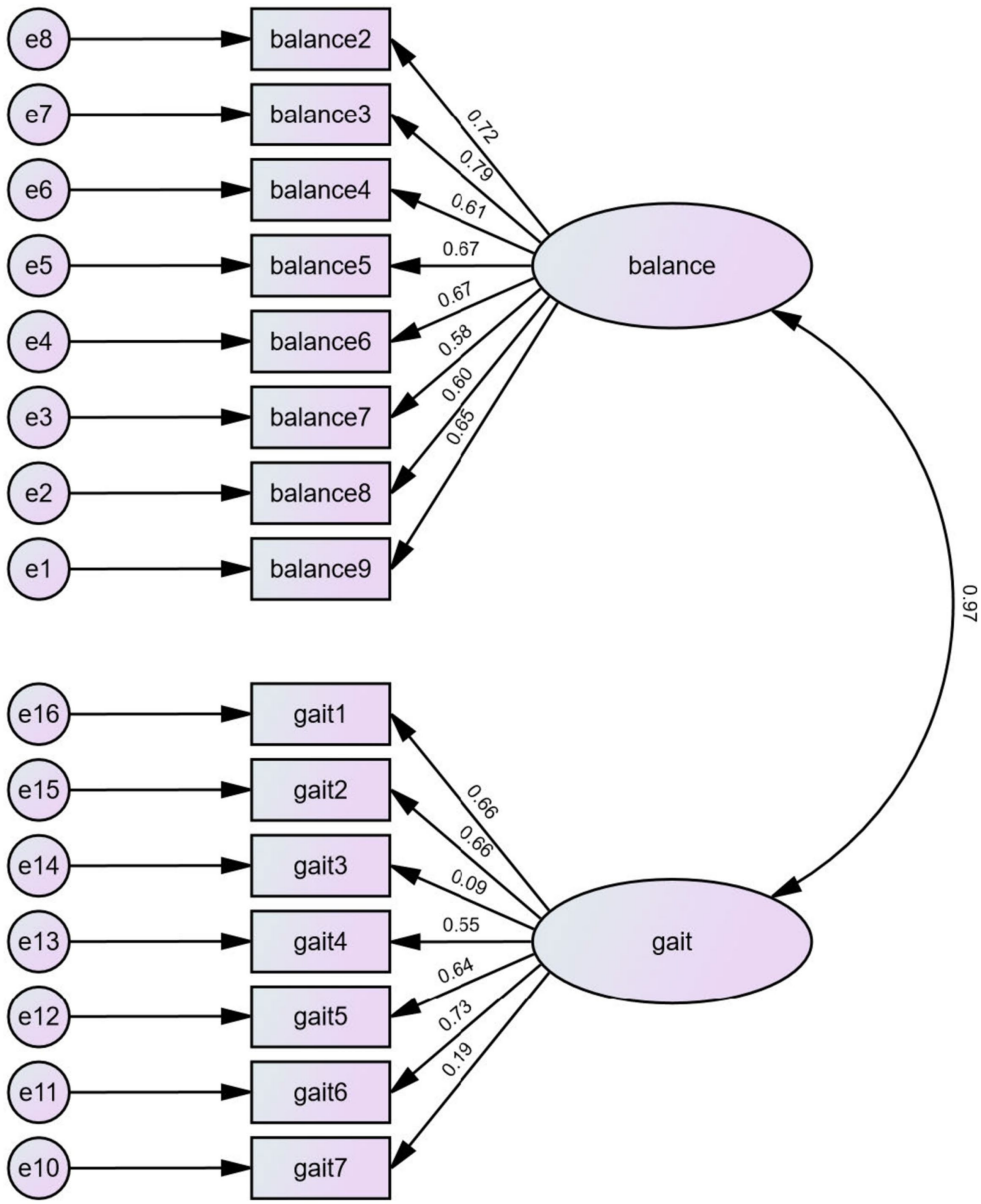
The maximum likelihood estimation.

The individual POMA items were entered into a confirmatory factor analysis with goodness-of-fit and maximum likelihood estimation to evaluate their content validity. The first question (item 1 for the balance subgroup) was excluded from the analysis for it had no variance, which all participants reported as “Steady, safe” and scored 1 on the item. As shown in Table 5, the two-factor model yielded a mediocre fit in the RMSEA model (0.095) and an acceptable fit in the CFI model (0.857) or the TLI model (0.831). An SRMR of 0.82 indicated a marginal fit. The CFA results suggested that the constructs of the two subscale components were acceptable. The maximum likelihood estimation is shown in Figure 2.

**Table 5:** Goodness-of-fit statistics for the two-factor models.

Fit model	Two-factor model
Chi (df)	149.407 (89)
RMSEA [90% CI]	0.095 [0.068 0.121]
AIC	211.407
BIC	283.660
CFI	0.857
TLI	0.831
SRMR	0.082

### 3.6 Construct validity

Finally, the Chinese POMA and its subscales were entered into the Pearson correlation coefficient with the BBS and TUG results to evaluate their construct validity. As shown in Table 6, the Chinese POMA and its subscales yielded strong positive correlations with the BBS and negative correlations with the TUG test, suggesting satisfactory construct validity.

**Table 6:** Correlation matrix of the Chinese POMA and subscales with BBS and TUGT.

	Berg Balance Scale		Timed Up and Go Test	
POMA Total T1	rs = 0.696	ps <0.001	rs = −0.752	ps <0.001
POMA Balance T1	rs = 0.762	ps <0.001	rs = −0.676	ps <0.001
POMA Gait T1	rs = 0.528	ps <0.001	rs = −0.757	ps <0.001

POMA Total T1, POMA Balance T1 and POMA Gait T1 refer to the total score of POMA, the balance and the gait subscale, respectively, measured on the first day of administration to inpatient treatment (*N* = 76).

## 4 Discussion

The current study tested the reliability and validity of the Chinese version of the Performance-Oriented Mobility Assessment (POMA) among 76 patients with chronic stroke (mean age: 62.04 ± 9.76 years; 34.2% female) with similar procedures and material to previous studies (3, 6, 7). The test–retest reliability was assessed on the first and second days of patient administration for inpatient treatment by the same physiatrist, and the inter-rater reliability was assessed on the first and second days of patient discharge from inpatient treatment by two independent physiatrists. The construct validity was measured based on external correlations with the BBS and TUG on the first day of inpatient treatment following the POMA. The results suggested that the Chinese POMA obtained a good overall internal consistency between 0.875 and 0.901 across the trials but a moderate internal consistency of its two subscales (balance and gait) between 0.688 and 0.930. The Chinese POMA showed high reliability between the test–retest trials (ICC = 0.872 to 0.997), an overall MDC95% of one score, and high inter-rater consistency (ICC = 0.988). However, the confirmatory factor analysis suggested a mediocre fit for the two-factor model (balance and gait). Finally, the correlation between the Chinese POMA, BBS, and TUG yielded satisfactory construct validity with moderate to high R coefficients. The details of this process are discussed below.

The overall internal consistency of the Chinese POMA among patients with stroke was good (0.901), consistent with the Turkish (0.88) (6) and Persian (0.94) versions of the POMA (7). Consistency may vary depending on stroke severity. Some motor functions were influenced more among some patients than others, which caused minor inconsistencies between the items. The internal consistency of the POMA in measuring mobility (balance and gait) in patients with stroke has not been reported in a previous reliability study of the original English version (3). Consequently, this study suggests a preliminary result that the Chinese POMA has good internal consistency. Further investigation is required to replicate the internal consistency of the POMA in both Chinese and the original English, measuring mobility (balance and gait) in patients with chronic stroke.

The Chinese POMA obtained good test–retest reliability (POMA overall ICC = 0.997; Balance ICC = 0.997; Gait ICC = 0.993), which is consistent with Turkish (POMA overall ICC = 0.94; Balance ICC = 0.88; Gait ICC = 0.92) and Persian versions of the POMA (POMA overall ICC = 0.97; Balance ICC = 0.95; Gait ICC = 0.96) (6, 7). The Chinese POMA also obtained good inter-rater reliability (POMA overall ICC = 0.996; Balance ICC = 0.999; Gait ICC = 0.988), which is consistent with Turkish (POMA overall ICC = 0.86; Balance ICC = 0.86; Gait ICC = 0.80) and Persian versions of the POMA (POMA overall ICC = 0.92; Balance ICC = 0.90; Gait ICC = 0.90) (6, 7). However, the overall MDC95% of the Chinese POMA was 1 with a standard deviation of 5.4, which was much smaller than the previously estimated English POMA among patients with stroke (3) of 6.0, with a standard deviation of 5.2 and also smaller than the Persian version of 3, with a standard deviation of 6.23 (7). Theoretically, MDC95% should mean a statistical meaning that patients rated the number of points not due to chance but due to an actual change in performance (26). The current results for MDC95% at 1 point should be interpreted with caution because we reported a high ICC of 0.997, which resulted in a very low standard error and led to a low MDC95%. Therefore, the results were likely caused by a bias in the statistical numbers despite all other results being comparable to those of reliability studies in different languages. Future studies should clarify this point by including larger sample sizes.

The construct validity measured using CFA showed a mediocre fit for the current two-factor model (balance and gait). This result is also in line with the previous Persian translation of the POMA (7), although the Turkish validation study did not conduct a factor analysis. The original development of the POMA did not engage in any factor analysis in the first place (4, 5), in which the factors of balance and gait were more theory-based than data-based. However, as Moulodi, Azad (7) suggested, the purpose of the POMA scale reliability is not to investigate a data-driven model with statistical figures but to determine whether it measures mobility. Future studies could investigate more fitted models to evaluate the POMA.

Finally, the Chinese POMA showed moderate-to-high content validity in correlation with BBS (POMA overall rs = 0.696; Balance rs = 0.762; Gait rs = 0.528) and TUG scores (POMA overall rs = −0.752; Balance rs = −0.676; Gait rs = −0.757), similar with Turkish (BBS: POMA overall rs = 0.866; Balance rs = 0.840; Gait rs = 0.770; TUG: POMA overall rs = −0.759; Balance rs = −0.675; Gait rs = −0.772) and Persian versions of the POMA (BBS: POMA overall rs = 0.90; Balance rs = 0.89; Gait rs = 0.85; TUG: POMA overall rs = −0.75; Balance rs = −0.73; Gait rs = −0.73) (6, 7). This is consistent with the results of testing the validity of the original English POMA in patients with stroke (3). This result suggests that the Chinese POMA could provide a valid prediction of fall risk as well as other similar measurements.

This study had two important limitations. First, we included a limited sample size of 76 patients with chronic stroke. Validation studies of questionnaires generally require a sample of 5 or 10 times the number of items, which should be 80 in our case. This number was first reached but dropped 4 patients meeting exclusion criteria. From the statistical approach, the current results obtained high correlation coefficients (*r* > 0.9), indicating the high statistical power of the tests with a larger sample size than the estimated minimum (14, 15). All available patients met our criteria during the past 2 years. However, 76 patients with stroke may not be enough to be generalized to all chronic stroke populations in terms of their characteristics, such as age (mean = 62.04 ± 9.76 years) and sex (34.2% female). Therefore, this study could not address solid conclusions on whether the POMA is valid among generalised patients with stroke or suggests a minimum score to detect performance changes, which requires future investigation. Second, as explained in the Methods section, considering clinical practice and ethics, the current study assessed inter-rater reliability after the patients were discharged from inpatient treatment. Consequently, the trial reflected post-treatment inter-rater reliability among patients with stroke, although it may diverge from pre-treatment inter-rater reliability. Although a previous study did not raise such a concern with a similar design (3), it would be better to clarify the pretreatment inter-rater reliability of the Chinese POMA in future studies.

In summary, this study examined the reliability and validity of the Chinese version of the POMA in patients with chronic stroke. The results demonstrated good internal consistency, test–retest reliability, inter-rater reliability, and content validity, supported by external correlations. However, the two-factor model of the balance and gait subscale structure revealed only moderate fit. Given these limitations, a cautious conclusion is warranted, defining the reliability of the Chinese POMA within the specific scope of the patient population tested. Future research should focus on further exploring the factorial structure, minimal detectable change (MDC95%), and pre-treatment inter-rater reliability using a more generalized and diverse sample of chronic stroke patients.

## Data Availability

The raw data supporting the conclusions of this article will be made available by the authors, without undue reservation.
