# Supplementary material for: Evaluating the reliability and validity of a Chinese version of the performance-oriented mobility assessment among patients with chronic stroke
Source: Front Neurol. 2024 Nov 13;15:1461069. doi: 10.3389/fneur.2024.1461069 (PMC11599243; doi:10.3389/fneur.2024.1461069)
Supplement: Supplementary file 1 [file Table_1.docx]

**Supplementary Materials**

**Contents**

[**English version of Performance-Oriented Mobility Assessment** 2](#_Toc181816430)

[**The Chinese version of Performance-Oriented Mobility Assessment** 5](#_Toc181816431)

# **English version of Performance-Oriented Mobility Assessment**

1. Balance

Initial instructions: Subject is seated in hard, armless chair. The following maneuvers are tested.

B1: Sitting Balance

0: Leans or slides in chair

1: Steady, safe

B2: Arises

0: Unable without help

1: Able, uses arms to help

2: Able without using arms

B3: Attempts to Arise

0: Unable without help

1: Able, > 1 attempt

2: Able to rise, 1 attempt

B4: Immediate Standing Balance (first 5 seconds)

0: Unsteady (swaggers, moves feet, trunk sway)

1: Steady but uses walker or other support

2: Steady without walker or other support

B5: Standing Balance

0: Unsteady

1: Steady but wide stance (medial heals > 4 inches apart) and uses cane or other support

2: Narrow stance without support

B6: Nudged (subject at maximum position with feet as close together as possible, examiner pushes lightly on subject’s sternum with palm of hand 3 times)

0: Begins to fall

1: Staggers, grabs, catches self

2: Steady

B7: Eyes Closed (at maximum position of item 6)

0: Unsteady

1: Steady

B8: Turing 360 Degrees

0: Discontinuous steps

1: Continuous steps

0: Unsteady (grabs, staggers)

1: Steady

B9: Sitting Down

0: Unsafe (misjudged distance, falls into chair)

1: Uses arms or not a smooth motion

2: Safe, smooth motion

1. Gait

Initial Instructions: Subject stands with examiner, walks down hallway or across room, first at “usual” pace, then back at “rapid, but safe” pace (using usual walking aids)

G1: Initiation of Gait

0: Any hesitancy or multiple attempts to start

1: No hesitancy

G2: Step Length and Height

Right swing foot:

0: Does not pass left stance foot with step

1: Passes left stance foot

0: Right foot does not clear floor completely (with step)

1: Right foot completely clears floor

Left swing foot:

0: Does not pass right stance foot with step

1: Passes right stance foot

0: Left foot does not clear floor completely with step

1: Left foot completely clears floor

G3: Step Symmetry

0: Right and left step length not equal (estimate)

1: Right and left step length appear equal

G4: Step Continuity

0: Stopping or discontinuity between steps

1: Steps appear continuous

G5: Path (estimated in relation to floor tiles, 12-inch diameter; observe excursion of 1 foot over about 10 ft. of the course)

0: Marked deviation

1: Mild/moderate deviation or uses walking aid

2: Straight without walking aid

G6: Trunk

0: Marked sway or uses walking aid

1: No sway but flexion of knees or back or spreads arms out while walking

2: No sway, no flexion, no use of arms, and no use of walking aid

G7: Walking Stance

0: Heels apart

1: Heels almost touching while walking

# **The Chinese version of Performance-Oriented Mobility Assessment**

**中文版Performance-Oriented Mobility Assessment**

一、平衡测试

B1患者坐在没有扶手的硬椅子上坐位平衡

0：斜靠或从椅子上滑下

1：稳定

B2 起身

0：没有帮助就无法完成

1：用上肢帮助才能完成

2：不用上肢帮助就能完成

B3 试图起身

0：没有帮助就无法完成

1：需要尝试1次以上才能完成

2：1次尝试就能完成

B4 立即站起来时平衡功能（站起抬头5秒）

0：不稳（摇晃，移动脚步，明显躯干摆动）

1：稳定，但是需要助行器或手杖，或抓住其他物体支撑

2：稳定，不需要助行器或手杖，或抓住其他物体支撑

B5站立平衡

0：不稳

1：稳定，但是两脚距离较宽【足跟中点间距离大于4英寸（1英寸=2.54cm）】，或使用手杖、助行器或其他支撑

2：稳定，两脚距离较窄，且不需要支撑

B7轻推（患者双脚尽可能靠拢站立，用手轻推3次）

0：开始就会摔倒

1：摇晃并要抓东西

2：稳定

B7 闭眼（同第6姿势）

0：不稳

1：稳定

B8 转身360°

0：不连续的脚步

1：不稳定（手臂及身体摇晃）

2：稳定

B9坐下:

0：不安全

1：用上肢帮助或动作不连贯

2：安全且动作连贯

二、步态测试

以舒适的速度，走3m

G1起步 得分

0：有迟疑，或须尝试多次才能启动

1：正常启动

G2抬脚高度和步长

a左脚跨步

0：脚拖地或太高大于1~2英寸

1：脚完全离地，但不超过1~2英寸

b右脚跨步

0：脚拖地或太高大于1~2英寸

1：脚完全离地，但不超过1~2英寸

c左脚跨步

0：跨步脚未超过站立的对侧脚

1：有超过站立的对侧脚

d右脚跨步

0：跨步脚未超过站立的对侧脚

1：有超过站立的对侧脚

G3步态对称性

0：两脚步长不等

1：两脚步长相等

G4 步伐的连续性

0：步伐之间不连续或中断

1：步伐连续

G5 走路路径（行走大约三公尺长）

0：明显偏移到某一方

1：轻度/中度便宜或使用步行辅具

2：走直线，且不需要辅具

G6 躯干稳定性

0：身体明显摇晃或需使用步行辅具

1：身体不摇晃，但需屈膝或有背痛张开双臂以维持平衡

2：身体不摇晃，无需屈膝、无背痛、不需张开双臂以维持平衡或使用辅具

G7 步宽（脚跟距离）

0：脚跟分开

1：走路时两脚几乎靠在一起

满分28分。其中平衡测试有9个项目，满分16分，步态测试共有7个项目，满分12分

如果得分少于24分，表示有平衡功能障碍；如果少于15分，表示有跌倒的危险性。
